# Electrochromic Inorganic Nanostructures with High Chromaticity and Superior Brightness

**DOI:** 10.1021/acs.nanolett.1c00904

**Published:** 2021-05-10

**Authors:** Marika Gugole, Oliver Olsson, Stefano Rossi, Magnus P. Jonsson, Andreas Dahlin

**Affiliations:** † Department of Chemistry and Chemical Engineering, Chalmers University of Technology, 41296 Gothenburg, Sweden; ‡ Laboratory of Organic Electronics, Department of Science and Technology, Linköping University, 60174 Norrköping, Sweden

**Keywords:** Electrochromism, Tungsten trioxide, Reflective displays, Fabry−Pérot cavity

## Abstract

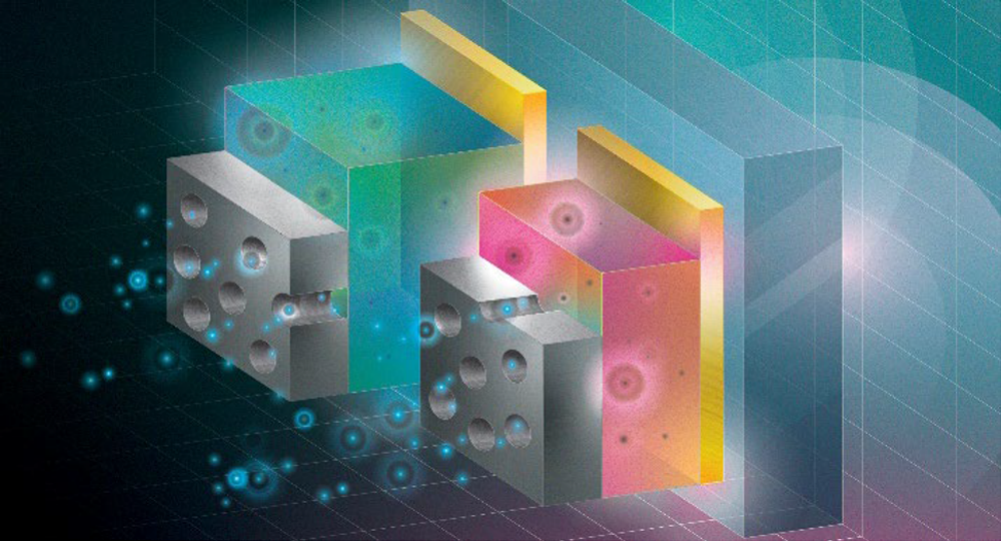

The possibility of actively controlling structural colors has recently attracted a lot of attention, in particular for new types of reflective displays (electronic paper). However, it has proven challenging to achieve good image quality in such devices, mainly because many subpixels are necessary and the semitransparent counter electrodes lower the total reflectance. Here we present an inorganic electrochromic nanostructure based on tungsten trioxide, gold, and a thin platinum mirror. The platinum reflector provides a wide color range and makes it possible to “reverse” the device design so that electrolyte and counter electrode can be placed behind the nanostructures with respect to the viewer. Importantly, this makes it possible to maintain high reflectance regardless of how the electrochemical cell is constructed. We show that our nanostructures clearly outperform the latest commercial color e-reader in terms of both color range and brightness.

## Introduction

Supported by major advances in nanofabrication techniques, there has been a renewed interest in structural colors based on solid state materials with nanoscale features. In nature, such colors emerge from dielectric materials,^1^ while humans have used metallic nanostructures supporting plasmon resonances for a long time.^2^ In the past few years, the possibility to actively control structural colors has attracted a lot of attention.^3−7^ Metallic nanostructures are particularly suitable for combining with electrochromic materials due to their conductive nature and have become highly interesting for various applications. In particular, reflective color displays or “electronic paper” technologies can offer great energy savings by replacing emissive displays (LCDs and LEDs) in various scenarios, especially under conditions of bright ambient light.^5^ Very recently, several fully inorganic nanostructures based on metals and tungsten trioxide (WO_3_) have been presented as strong candidates for durable electrochromic devices.^8−14^ In contrast to organic electrochromism based on conducting polymers,^15^ the use of WO_3_ does not cause swelling of the material, i.e., the optical tunability is solely a result of the change in permittivity of the material upon ion intercalation.^16^ Furthermore, WO_3_ has proven stable enough for use in commercial devices such as smart windows.^4^

However, existing electrochromic devices based on WO_3_ remain limited in terms of the main performance factors required for a reflective color display. The two most fundamental properties for good image quality are brightness (which translates to reflectance for electronic paper) and chromaticity (the color purity measured as the gamut in the CIE).^5^ Although many innovative and promising nanoscale designs have been presented recently,^4^ the colors may be limited to the green-red spectral region^11^ and/or the reflectance is low.^10^ In fact, the aspect of brightness is often neglected: if absolute reflectance values are presented at all, it is not explained properly how they were measured. (For instance, the type of reference reflector becomes critical.) Without achieving both a strong reflection and a broad color range, the scope of applications for electrochromic nanostructures will be limited, even when it comes to simple decorative purposes. Maintaining high brightness is particularly critical for electronic paper technologies since only a fraction of the display surface will show a given color when using subpixels arranged side by side.^5^ Because of this intensity loss, it is essential to find electrochromic surfaces that can provide many different colors as it can reduce the number of subpixels needed. In some cases, this can be achieved with polarization-dependent reflection of colors and liquid crystals,^17^ but such approaches still result in low absolute reflectance due to the requirement of polarizers.^18^ In addition, regardless of the type of electrochromic surface used, the reflectance is always further reduced in practice because a counter electrode is needed in the device design.^19^ This is typically achieved with minimally absorbing charge storing electrodes, which nevertheless eliminate a considerable fraction of the incident light.^20^

In this work, we show a new design for inorganic electrochromic nanostructures which circumvents the problem of low reflectance while still providing an excellent color range. This is achieved by selecting the right metals and reversing the thin film layers, enabling all electrical components to be “hidden” behind the reflective surface. The devices show excellent performance in terms of both chromaticity and reflectance. Also, the power consumption is ultralow because of bistability (coloration memory). Finally, we propose a dual multichromatic pixel layout (instead of the conventional RGB) that enables high image quality in a reflective color display and show that this clearly can outperform a state-of-the-art electronic reader in color in terms of both brightness and color quality.

## Results and Discussion

Inspired by recent work on tunable cavity resonances based on Li^+^ intercalation in WO_3_,^9−12,21^ we evaluated different nanostructures where WO_3_ was sandwiched between a mirror and a semitransparent metal film with nanoholes, generating a Fabry–Pérot cavity.^11,13,21−23^ Colloidal lithography and standard thin film deposition techniques were used, which enable easy production of large-area samples. We tested different metals for extending the color range while maintaining stability in electrolyte environments. We found that platinum was ideal as broadband back-reflector, while 20 nm gold was still ideal color-wise for the semitransparent nanohole layer.^13^ A ∼50 nm Pt mirror was sufficient to suppress transmission almost entirely due to the short penetration depth of incident light.^24^ Also, due to the inert nature of both Pt and Au, the devices were highly stable with respect to oxidation, both in the presence of oxygen and upon applying anodic potentials. Color variations over the surface originate from the sputter coater that deposits WO_3_ since it is designed for relatively small samples. The uniformity may be improved by using other deposition methods in the future.^25^

**Figure 1:**
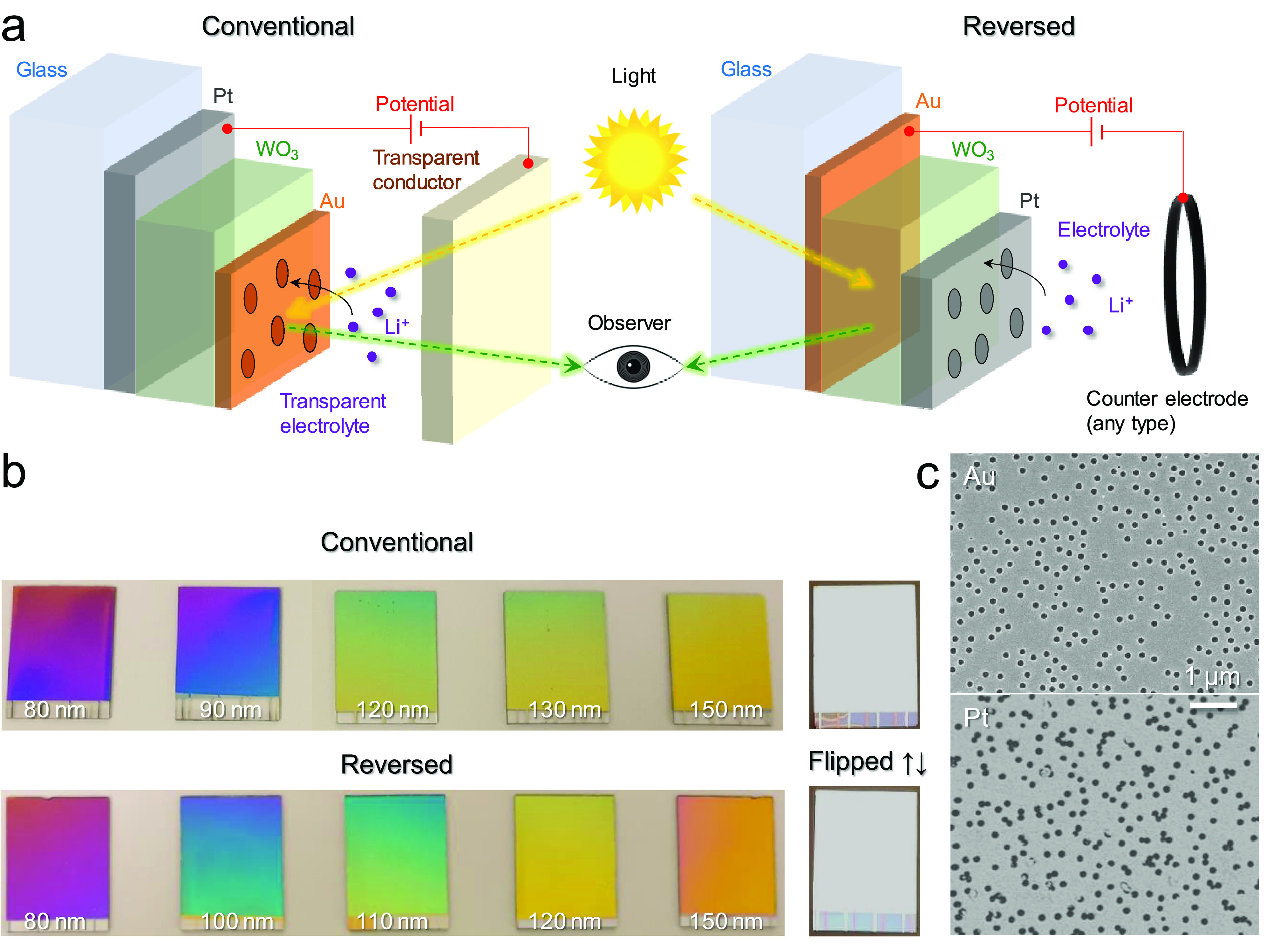
Nanostructure and device design. (a) Schematics illustrating the conventional and reverse architectures. (The sun and the eye are free clipart under the CC0 1.0 Universal Public Domain Dedication.) (b) Photos of samples (18 × 24 mm^2^) with different thickness of WO_3_. Samples placed with Pt reflector facing up are also shown for comparison. (c) Electron microscopy images showing nanoholes in Au (conventional) or Pt (reversed).

For electrochromism based on conductive polymers, it has been shown that ions required for switching coloration state can be transported through opaque porous materials.^26,27^ Inspired by such work, we altered the “conventional” device outline by introducing the nanoholes in the Pt film instead, leading to the “reversed” design (Figure 1a). The Au film was still 20 nm but without holes and if the absence/presence of nanoholes is ignored, the incident light experiences the same path through the thin films in both designs. One reason the reversed design is possible is the thin Pt film (up to 70 nm), which prevents the colloids from becoming buried after metal deposition. The colloids could therefore be removed by carefully scraping the surface.^28^ As a result, the observer can look directly onto the structural colors through a fully transparent solid support, which also offers good physical protection against scratches and contaminations. In the reversed design, the electrolyte and counter electrode can be of practically any size and material suitable for efficient electrochemical performance and long-term stability. In comparison, the conventional design requires that the observer looks through both electrolyte and counter electrode in the display device,^19^ which severely limits how the circuit can be configured and possible choices of materials. Just like the conventional samples, the reversed samples had practically no transmission (∼0.1%).

**Figure 2:**
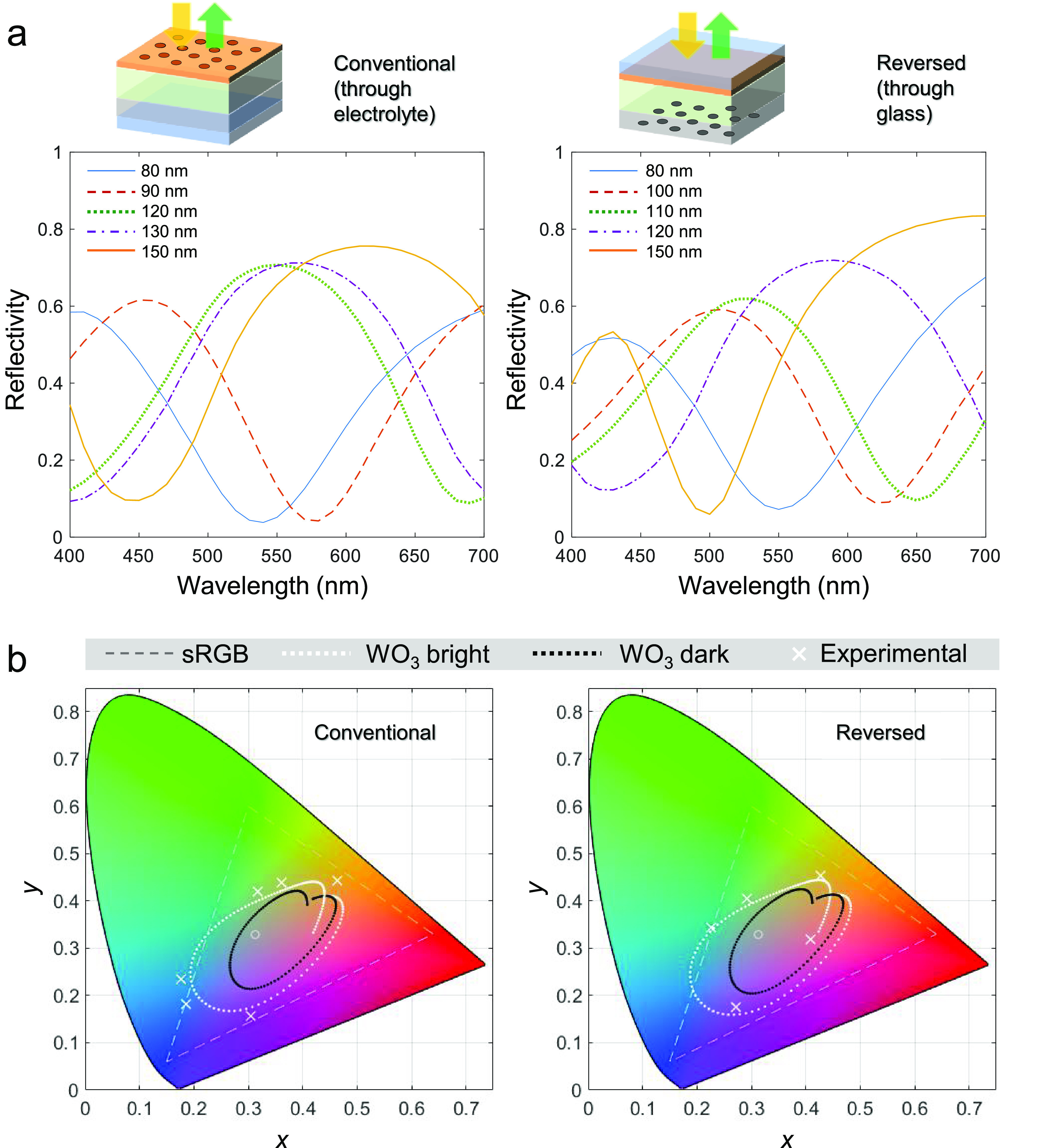
Reflectance and color range. (a) Absolute reflectance spectra measured under diffuse illumination for different WO_3_ thickness. (The conventional samples are immersed in a liquid which has the same refractive index as the electrolyte.) (b) Simulated color range in the CIE diagram for WO_3_ thickness from 20 to 150 nm (white dots) and experimental data points (crosses). The black dots show the simulated color range when WO_3_ is in its dark state. The circles indicate the D65 reference white point.

Importantly, we observed no significant differences in optical response for the conventional and reversed designs. All colors could be achieved in both designs by changing the WO_3_ thickness (Figure 1b). Furthermore, the nanohole arrays looked similar in electron microscopy images, although with some connected holes in Pt (Figure 1c). The reflectance (polarization insensitive) was high in absolute numbers, and the spectra were very similar as long as the WO_3_ thickness was the same (Figure 2a). We concluded that any difference in the far field response from the two designs is smaller than the inherent sample-to-sample variation, which is mainly determined by the precision with which the WO_3_ film thickness can be reproduced by sputtering (±10 nm). This implies that the nanoholes do not have a strong influence on the optical properties; i.e., the Fabry–Pérot cavity resonance is almost entirely responsible for the coloration. This differs from previous work with Al_2_O_3_ as the cavity, which gives rise to plasmonic activity that contributes to the structural colors.^13,22,23^ Dark field imaging showed only weak red scattering from the nanoholes in Au and practically no scattering from holes in Pt (Figure S1). Control samples without any nanoholes exhibited similar (within experimental variation) reflectance spectra in the visible (Figure S2). As another control, changing hole diameter did not lead to any significant changes in the reflectance spectrum (Figure S3). Thus, the purpose of the nanoholes (in Au or Pt) is to allow transport of Li^+^ ions to the WO_3_ as shown by Hopmann et al.^11^ However, finite difference time domain (FDTD) simulations on a square lattice^29^ did suggest plasmonic activity in the near-infrared for the conventional design (see Figure S4 and related discussion).

Due to the negligible contribution from plasmonic activity to the spectra, they were in good agreement with simple Fresnel simulations of the thin film system without nanoholes (Figure S5). Literature data was used for the permittivity of Au,^30^ Pt,^24^ and WO_3_^16^ (either the bright or the dark state). To estimate the total color range that can be obtained, we simulated the CIE coordinates generated by small stepwise changes in WO_3_ thickness, resulting in lines in the *xy* chromaticity diagram representing a wide gamut around the white point (Figure 2b). Remarkably, most experimental data points appeared *outside* of the simulated gamut and some even outside the standard RGB range. The discrepancy between experiments and simulations is mainly due to minor differences in the permittivity of our WO_3_ films^13^ compared to literature data^16^ (rather than the presence of the nanoholes). Simulating the cavities with WO_3_ in its absorbing state leads to a smaller gamut (Figure 2b), which is due to the broadened spectral features. Nevertheless, a full color range is still generated. Increasing the thickness of WO_3_ above ∼150 nm did not extend the color range because multiple reflectance peaks start to appear within the visible, which is not beneficial for chromaticity for these nanostructures. Similarly, decreasing the thickness to tens of nanometers reduces color quality because the optical path length becomes too short. We note, however, that further improvements may be possible by double cavity structures.^21^ The viewing angle effect was negligible for incident angles <10° after which the colors changed only slightly (see simulations in Figure S6 and the video in the Supporting Information showing tilting of a sample).

**Figure 3:**
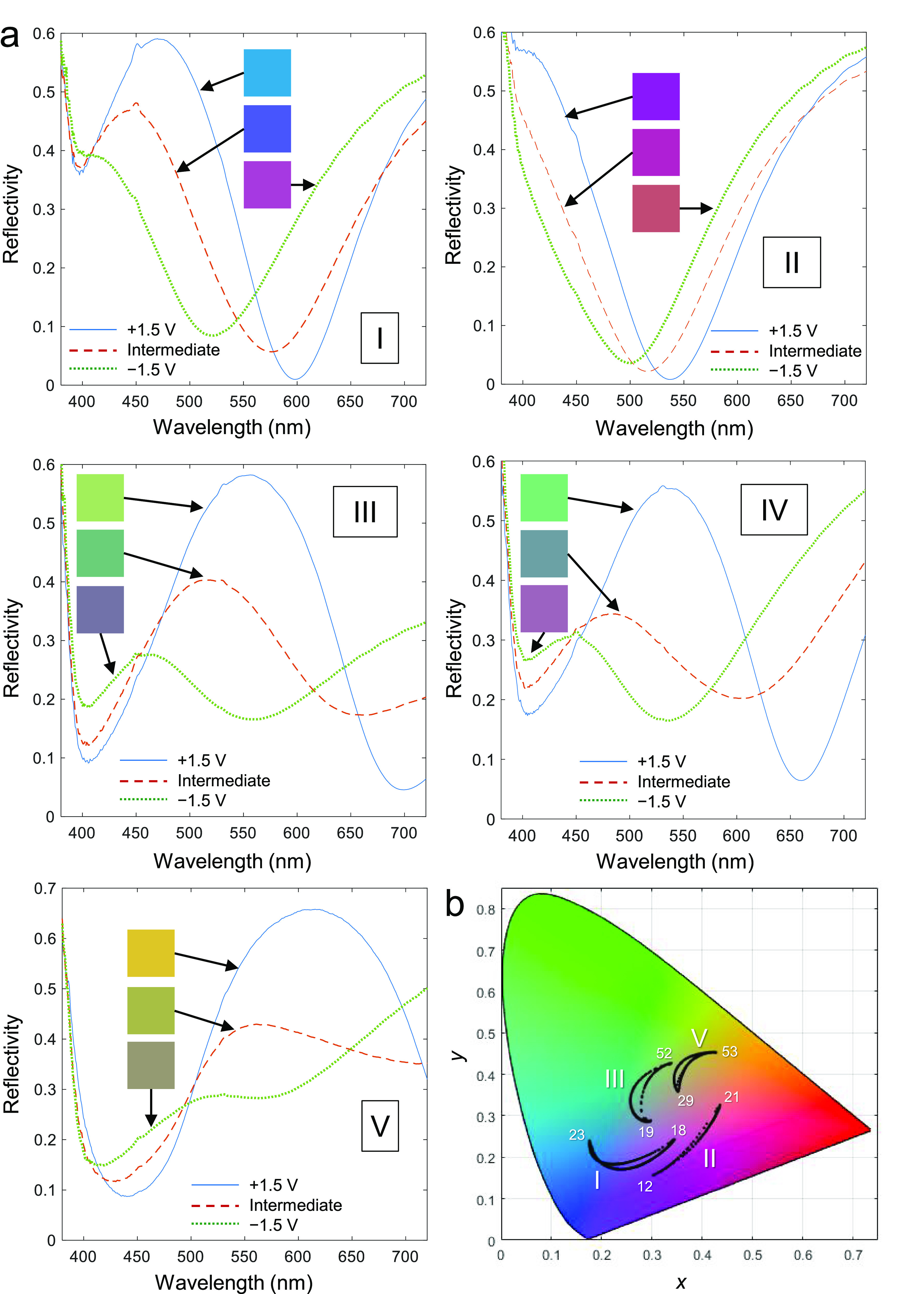
Electrochemical color tuning. (a) Examples of reflectance changes when switching voltage between −1.5 and +1.5 V vs (Ag/Ag^+^). I: 100 nm WO_3_ (conventional). II: 80 nm WO_3_ (conventional). III: 120 nm WO_3_ (conventional). IV: 110 nm WO_3_ (reversed). V: 120 nm WO_3_ (reversed). In all cases, an example of an intermediate color state is shown. Note that in these experiments, the illumination light passes next to the Pt counter electrode for the conventional design. This enables a proper comparison with the reversed design, but will not be possible in a real flat panel display device. The squares show pseudo colors based on RGB values obtained from the CIE coordinates. (b) Corresponding movement in the CIE *xy* diagram. The numbers are the *Y* values at equilibrium for the end voltages. For all samples except III the higher *Y* corresponds to the bright state of the WO_3_. (Sample IV is not included since it is similar to III.)

In order to dynamically tune the colors, a potential was applied to the nanostructures (ranging from +1.5 to −1.5 V vs Ag/Ag^+^) and the reflectance was monitored during Li^+^ intercalation.^9−11,13^ For the reversed device design, the incident light illuminated the backside of the sample instead of going through the electrochemical cell. Figure 3 shows examples of color changes in terms of spectra and CIE coordinates. In brief, the reflectance maximum for the fundamental cavity mode moves across the visible due to changes in the permittivity of WO_3_. There is once more almost identical spectra for the conventional and reversed designs if the WO_3_ thickness is similar (compare samples III and IV). Notably, even in the absorbing state when Li^+^ is intercalated, the reflectance remains quite high (maximum >50%). For electrochromic applications, the brightness should also preferably be quantified in a manner that accounts for the wavelength dependent sensitivity of the eye (the luminosity function). Therefore, we also calculated the CIE *Y* value of the samples in their different coloration states. To establish some reference values for this parameter, a perfect broadband mirror has *Y* = 100, while we measured *Y* = 63 for white regions on a common newspaper and *Y* = 84 for an ordinary A4 paper (a very high value due to optical brightening agents). With black ink printed, the newspaper was reduced to *Y* = 9 and the printer paper was reduced to *Y* = 5 (spectra in Figure S7). During Li^+^ intercalation, *Y* generally decreased due to higher WO_3_ absorption (see values in Figure 3b), but occasionally increased when the voltage was lowered if the color moved closer to green; i.e., the “bright” WO_3_ state is not necessarily the brightest state of the nanostructure for the viewer. This is because the luminosity function peaks at 560 nm and this result illustrates how the *Y* parameter is an important complement to the reflectance values. The small hysteresis during switching is associated with the abrupt reversal of voltage and, most likely, a heterogeneous distribution of Li^+^ throughout the WO_3_ film.

The switching dynamics showed that a complete switch took ∼1 min (see videos in the Supporting Information), which is slightly slower than for a WO_3_ film in direct contact with the electrolyte.^13^ This is expected because the nanohole array slightly hampers the transport of Li^+^ ions. At the same time, the holes are necessary for tuning the cavity resonance (control samples without holes could not be switched). Furthermore, thicker WO_3_ films took longer time to switch, which is expected since Li^+^ needs to be transported through the whole layer. The speed can likely be improved by slightly increasing the amount of nanoholes or by altering the porosity of the WO_3_,^25^ but it is already sufficient for display applications where images do not necessarily need to be frequently updated (e.g., advertisement or decorative images). The coloration state is determined by the degree of Li^+^ intercalation, which has an equilibrium state determined by the applied voltage. However, we always applied the same voltages since any intermediate coloration state could be maintained simply by aborting the switch; i.e., the electrochromic nanostructures exhibited bistability for several minutes (Figure S8). This means that the power consumption is essentially zero for maintaining any selected color throughout the switch and only a low amount of energy is required for changing coloration state^13^ (at the most 40 mJ/cm^2^ for a full switch of the thickest WO_3_).

**Figure 4:**
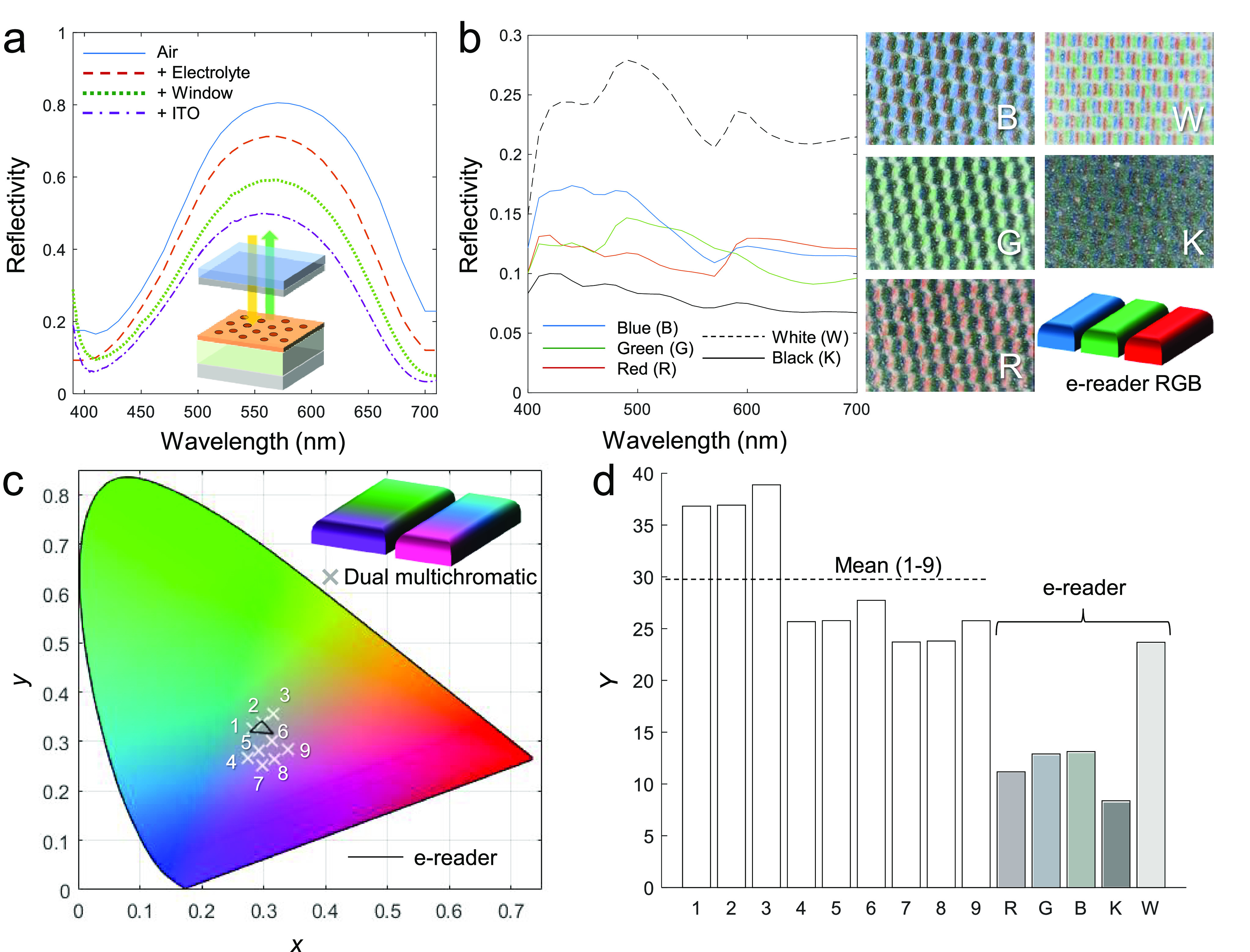
Outperforming the latest commercial color e-reader. (a) Characteristic intensity losses (reflectance decrease) when electrolyte and counter electrode are introduced in a conventional cell design for a flat panel display device. (b) Reflectance of different colors from the latest commercial color e-reader. The corresponding images shows the pixels viewed under a microscope (images show 2.2 × 1.5 mm^2^). (c) Example of colors that can be generated by a dual multichromatic subpixel outline (assuming 90% fill fraction for the display surface). For comparison, the RGB colors of the commercial e-reader are included. (d) Brightness comparison in terms of *Y* values for the dual multichromatic pixel layout compared to the e-reader RGB.

We emphasize that the reflectance values we obtain can be maintained in real devices because of the reversed design, which is not influenced by how the electrochemical circuit is constructed behind the nanostructures. In order to quantitatively show how important the reversed design is, we measured the reflectance loss for a sample in the conventional design using a vertical cell configuration with an indium tin oxide (ITO) coated glass cover. First, introducing the electrolyte leads to a small reflectance decrease (Figure 4a). Next, the loss in reflectance is an additional 20% when introducing a glass window and an ITO coating on this glass. (The corresponding loss in *Y* is from 74 to 44.) Furthermore, the ITO film (130 nm thick and with the same area as the nanostructure) did not have sufficient charge storage capacity to completely switch the WO_3_. This means that its effective capacitance needs to be increased, which is far from straightforward. One option is to introduce a minimally coloring charge storing polymer, but this leads to further intensity losses of tens of percent,^20^ especially since light passes through twice. Commercial WO_3_ devices often use nickel oxide as the counter electrode as it is anodically coloring, but the non-negligible absorption in its “bright” state leads to similar intensity losses.^31^ Thus, in total, the reflectance is severe reduced (approximately cut in half) when a functioning flat-panel display device is constructed using the conventional approach. In contrast, our reversed design suffers no intensity losses at all since neither the electrolyte nor the counter electrode is in the light path (Figure 1a). There is also room for further improvements: the reflectance can become even higher on lower refractive index supports such as Teflon.^32^ Fresnel calculations and experiments showed that the nonresonant absorption increases slightly when the gold film is in contact with a higher refractive index medium. (Even for a single metallic interface, the refractive index of the environment influences the effective skin depth and the absorption.) Also, antireflection coatings can be introduced on the other side of the solid support facing the viewer to further reduce the small reflection at this additional interface.

Because of the excellent brightness and chromaticity of our nanostructures, we hypothesized that they would outperform existing electronic readers in color. To quantitatively investigate this, we first performed measurements on the latest such device on the market at the time of writing: the PocketBook Color, which is an electrophoretic display^33^ that uses color filters to generate RGB subpixels using E-ink Kaleido technology. Reflectance spectra of the PocketBook showing the primary colors as well as white/black (all subpixels on/off) showed very low brightness (Figure 4b). This is not surprising considering that the reflectance of each electrophoretic cell is <50%, which is to be divided by more than a factor of 3 when subpixels are introduced.^13^ Note that in order to ensure capturing all light from the e-reader, we measured with an integrated sphere that simulates ambient illumination and includes nonspecular reflection.

In contrast to RGB subpixels that are tuned in intensity, the electrochromic inorganic nanostructures truly alter their color; i.e., they move in the CIE diagram (Figure 3b). This opens up for new ways to introduce subpixels and perform color mixing by testing linear combinations of reflectance spectra at different voltages for some selected values of WO_3_ thickness. This quickly becomes a complex multiparameter optimization process. Our main point here is that although optimization with respect to color purity is important and has been investigated in depth,^34^ brightness should not be neglected. If a nanostructure would reflect only a very narrow wavelength band (∼10 nm), it will represent superb color quality, but even if the reflectance is 100% at the resonant wavelength, the visibility will still be poor since only a very small fraction of the incident white light is reflected. Therefore, the *Y* value, which averages over the whole visible, is a better parameter than the maximum reflectance for representing brightness. We will simply present an example to illustrate that our electrochromic nanostructures can easily outperform the commercial device in both respects, especially because of the reversed configuration. We show a dual pixel design assuming a display area covered to 35% by nanostructures with 90 nm WO_3_ and to 55% by nanostructures with 110 nm WO_3_. The remaining 10% represents area needed for pixel separation and is assumed to have a flat reflectance spectrum of 50%. (The pixel fill fraction is thus assumed to be 90%, similar to that in commercial devices.) The reflectance spectra are generated by taking different coloration states for the two pixels with weight factors 0.35 and 0.55 (plus 0.1 with 50% reflection). We selected the reversed design for both subpixels because it is the most relevant to implement as it will not suffer from intensity losses in a flat panel display. (Very similar results were achieved for the conventional design, but only if the effect from the counter electrode as in Figure 4a was ignored.) Figure 4c shows nine examples of colors in the CIE diagram that can be generated by selecting different coloration states for the two pixels. For comparison, the much smaller color range of the commercial e-reader is also shown. Finally, we compare the *Y* values of our different colors with the e-reader, showing a strong enhancement also in terms of brightness (Figure 4d). The mean value for the example colors generated by the dual pixel design is *Y* = 29, which is almost three times higher than for the primary colors of the e-reader. Furthermore, all 9 colors have a *Y* value which is higher even than the fully “white” state of the e-reader. The dual pixels design can also in principle give different grayscale states by selecting different pairs of Li^+^ intercalation levels, one for each metasurface, which all end up around the CIE white point when combined. This is similar to the proposed “universal pixel” that can alter both brightness and hue,^6^ although further improvements are necessary to reach this goal.

## Conclusions

We have shown a new design for electrochromic inorganic nanostructures based on cavity resonances in WO_3_ where a thin Pt film is used as the mirror. Besides providing a broad color range and high reflectance, the structures enable us to reverse the configuration in electrochromic devices so that the electrochemical cell ends up behind the reflective surface. This makes it possible to maintain high brightness regardless of electrolyte and counter electrode, which otherwise typically reduce the brightness by at least a factor of 2. Furthermore, the nanostructures alter their color so much upon Li^+^ intercalation that two different structures are sufficient to generate all colors. Hence, we propose a dual multichromatic subpixel layout in order to improve brightness compared to RGB subpixels. This work is the first clear demonstration of how electrochromic nanostructures outperform the latest commercial product of the market in terms of both chromaticity and brightness. Our nanostructure design enables new possibilities for electronic paper in color because of the improved image quality, which should be sufficient for the technology to replace energy costly emissive displays in many scenarios. In the future, our results may also be relevant for “truly solid state” electrochromic devices that rely on ambient humidity.^35^

## Supporting Information Available

Experimental section, video descriptions, figures showing dark field images, additional reflectance spectra, FDTD simulations, comparison of Fresnel models with experimental spectra, bistability test and simulated viewing angle dependence (PDF)
Switching in the conventional design (MP4)
Switching in the reverse design (MP4)
Angular dependence (MP4)
