# Supplementary material for: Electrochromic Inorganic Nanostructures with High Chromaticity and Superior Brightness
Source: Nano Lett. 2021 May 10;21(10):4343–50. doi: 10.1021/acs.nanolett.1c00904 (PMC8289301; doi:10.1021/acs.nanolett.1c00904)
Supplement: Supplementary file 1 — nl1c00904_si_001.pdf [file nl1c00904_si_001.pdf]

Electrochromic Inorganic Nanostructures with High Chromaticity and Superior Brightness

*Marika Gugole,[1] Oliver Olsson,[1] Stefano Rossi,[2] Magnus P. Jonsson[2] and Andreas Dahlin.[1]**

1 Department of Chemistry and Chemical Engineering, Chalmers University of Technology, 41296 Gothenburg, Sweden.

2 Laboratory of Organic Electronics, Department of Science and Technology, Linköping University, 60174 Norrköping, Sweden.

* Corresponding author: adahlin@chalmers.se

Brief description of videos

Video 1: Switching in the conventional design. Colors covered are green, yellow and blue.

Video 2: Switching in the reverse design. Colors covered are blue, magenta and red.

Video 3: Angular dependence, showing a sample which is tilted up to 50°.

Experimental

Materials: Propylene carbonate and poly(ethylene glycol) was purchased from Sigma. $LiClO_4$ (anhydrous) was purchased from Fischer Scientific. ITO glass was purchased from Naranjo substrates. For analyzing paper, a local newspaper (Expressen) was purchased and a black box was printed (Kyocera TASKalfa 356ci) on ordinary A4 paper (New Future Multi).

Nanofabrication: Supports were borosilicate microscopy cover glasses. Au and Pt films were deposited by physical vapor deposition (Lesker PVD 225). For the conventional design, a 5 nm Ti layer was included under Pt and a 1 nm Ti layer was included under Au to promote adhesion. For the reverse design, 1 nm Ti was used as adhesion between Au and glass. WO_3 was deposited by reactive sputtering (Nordiko 2000) with 32 sccm Ar and 8 sccm O_2 at 20 mTorr and 150 W. The WO_3 thicknesses was estimated by a spectroscopic ellipsometer (J.A.Wollam M2000). Colloidal lithography was performed on WO_3 using 147 nm polystyrene colloids (Microparticles). For the reversed samples, colloids were removed by carefully scraping the surface instead of using tape.[1]

Electrochemical measurements: A commercial liquid cell (RedoxMe) with an Ag/Ag^+ reference electrode and Pt counter electrode was used for three-electrode measurements with a potentiostat (Gamry Interface 1000/1010). When testing ITO, this material acted as both counter and reference electrode. All switching was performed in propylene carbonate with 1 M $LiClO_4$.

Optical measurements: The reflectance spectra were measured using diffuse illumination by a CM-700d spectrophotometer (Konika Minolta) and light collection at 8° against the surface normal. When measuring spectra of conventional samples (without doing any switching), poly(ethylene glycol) was mixed with milli-Q water in order to increase the refractive index to be the same as for propylene carbonate. To measure spectra during electrochemical switching, a home-built microspectroscopy setup was used.[2] The illumination and collection was with a 4× air

---

[1] Xiong et al. *Analyst* **2016**, 141 (12), 3803-3810.

[2] Gugole et al. *ACS Photonics* **2020**, 7 (7), 1762-1772.

objective (NA 0.10) and optical fibers were connected to the lamp (100 W tungsten) and the spectrometer (B&WTek photodiode array). When testing samples in the conventional design, light passed through a glass window and next to the Ag and Pt electrodes (see video). A broadband dielectric mirror (BB05-E02, Thorlabs) was used to measure the reference intensity. In order to ensure accurate absolute reflectance, the mirror used to obtain reference intensity was also measured in the CM-700d instrument.

Pictures: Photos of samples were taken with a Samsung S9 phone with standard settings. The commercial e-reader pixels were visualized with a digital handheld microscope. The different colors were measured by assigning their sRGB values when drawing in PowerPoint, after which the document was exported as a pdf file and transferred to the e-reader for viewing and measuring. Dark field images were recorded with an Axiocam506 color camera in a microscope (Zeiss Axio Observer 7) with a 50× air objective designed for dark field illumination in reflection mode.

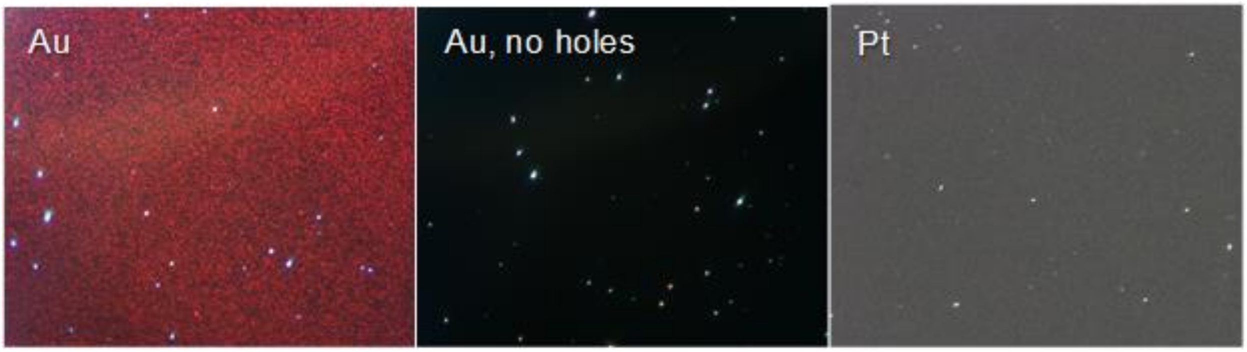

Figure S1 Dark field images showing weak deep red light scattering from nanoholes in Au. There is no significant scattering in the absence of holes. For holes in Pt the scattering is barely detectable and has no characteristic color.

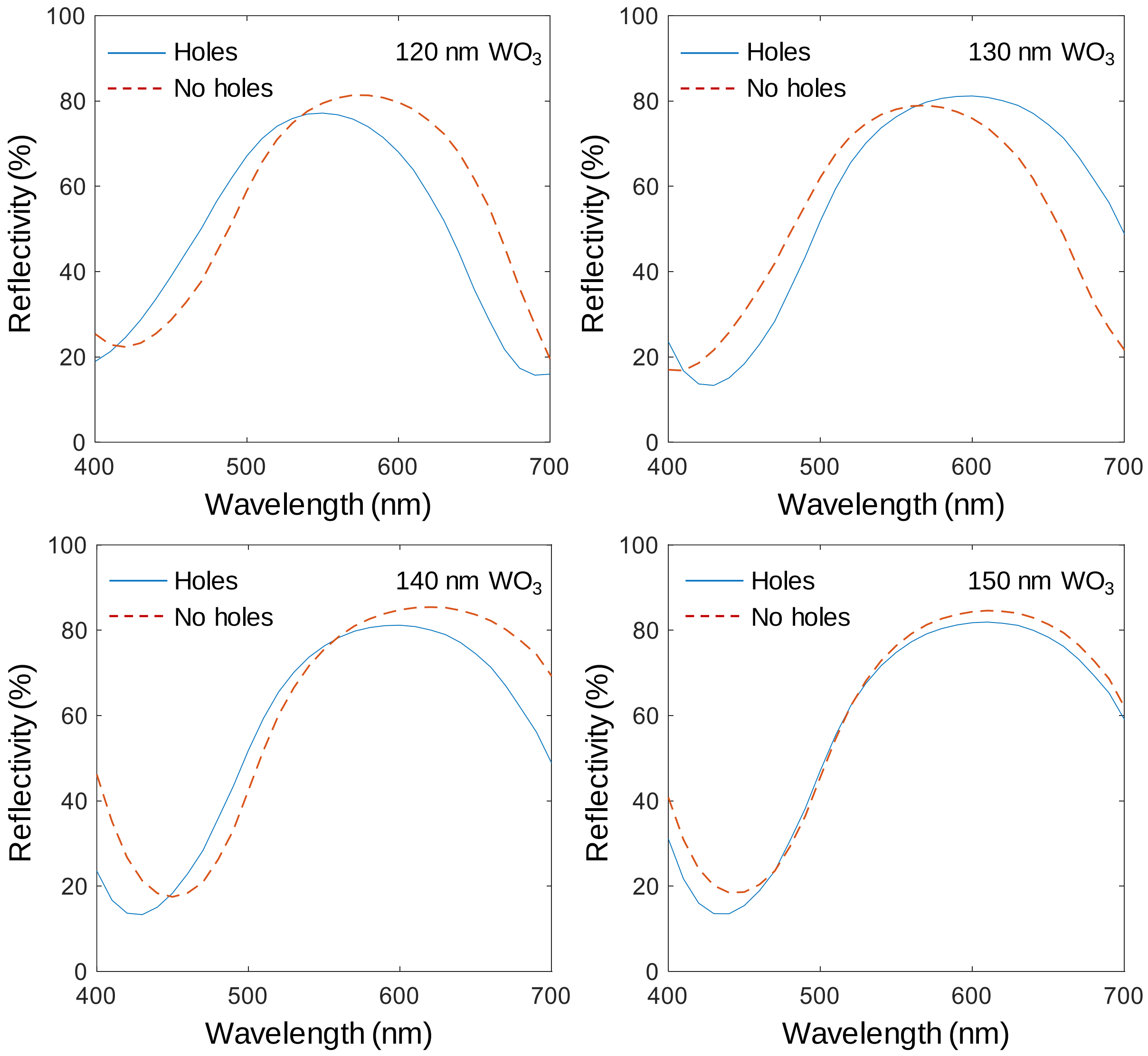

Figure S2 Reflectance spectra of samples made by the conventional design but without nanoholes in the gold film. The spectral changes compared to having holes are not significantly larger than the inherent sample to sample variation.

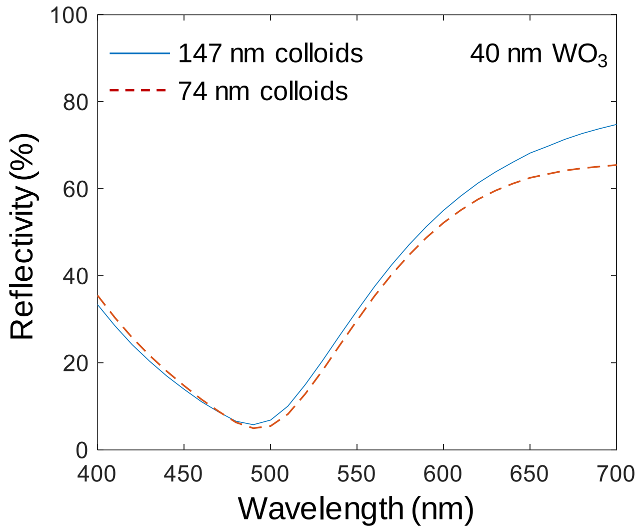

Figure S3 Reflectance spectra of samples with different diameter of nanoholes (in Au). As in the previous figure, the spectral changes are not significant.

Numerical simulations

Simulations were preformed to find out if the lack of plasmonic activity in the visible is because the resonances are shifted to the NIR or if they are too damped. FDTD calculations in Lumerical were used similarly to previous work.[3] Periodic boundaries were used to model a square lattice in the surface plane. A perfectly matched layer was used in the direction perpendicular to the surface and the span of the FDTD simulation was 600 nm. The minimum mesh step was 2 nm, auto-non-uniform with a 2 nm mesh override over the nanoholes. The electric and magnetic field were recorded in continuous wave normalization, meaning that the fields are normalized by the Fourier transform of the source pulse.

The simulations confirm that the nanoholes do not significantly influence the spectra, at least not in the visible. For the conventional design, a clear minimum in reflectance appears just above 1000 nm when an array of nanoholes is present in Au. We attribute this reflectivity minimum to coupling to surface plasmons since it does not appear when there are no nanoholes in Au. The resonance appears in the NIR because the WO_3 layer has high refractive index compared to other dielectrics that can be sandwiched between the metals (such as Al_2O_3). For the reversed design, no reflectance dip appears. This is probably because of less efficient coupling to plasmons when the holes are in Pt which is more absorptive than Au.

[3] Malekian et al. *Nanoscale Advances* **2019**, 1 (11), 4282-4289.

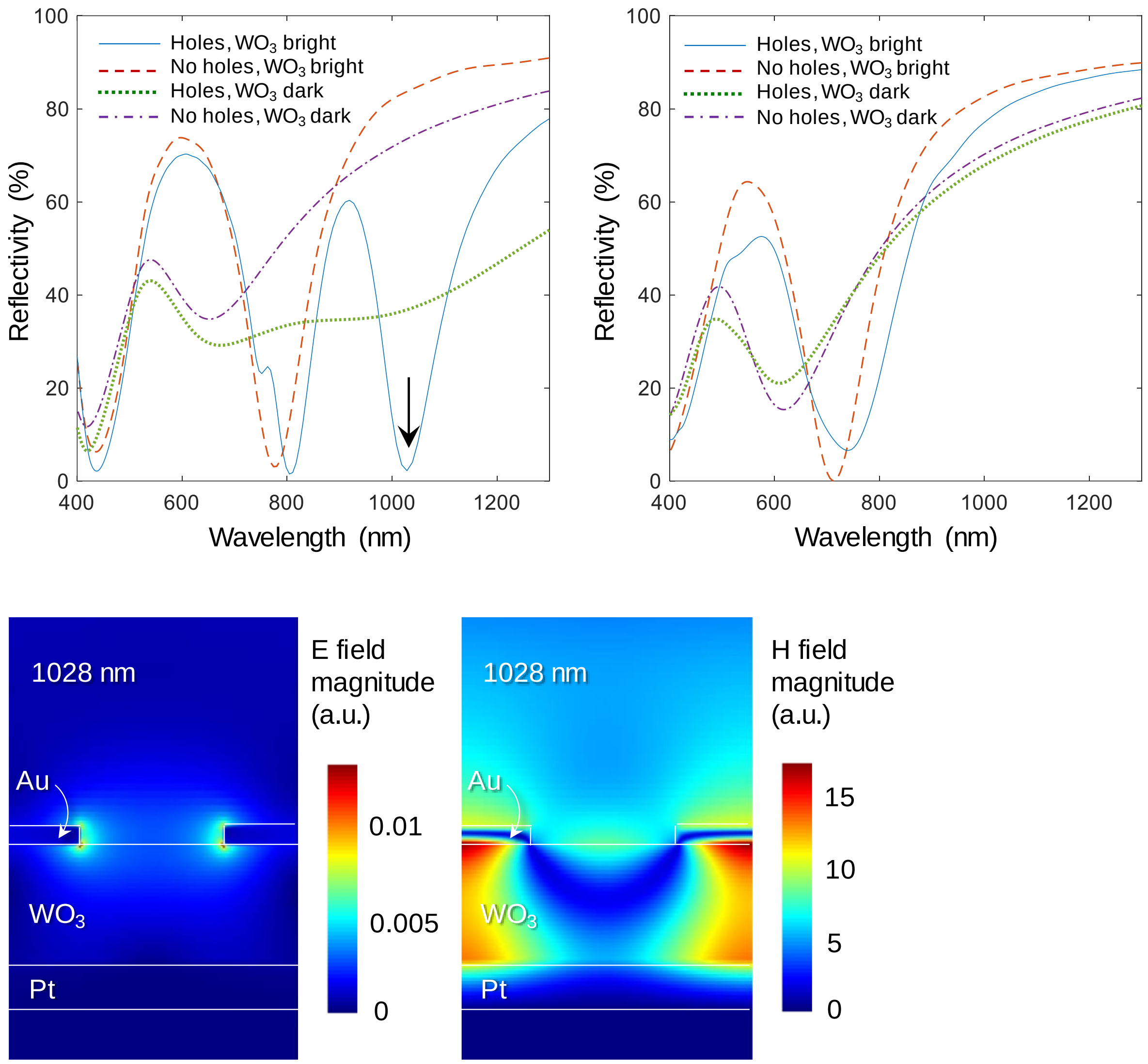

Figure S4 FDTD simulations of the reflectance from the nanostructures with or without nanoholes. Left shows conventional, right shows reversed. The WO_3 thickness is 130 nm and the hole diameter is 150 nm. The electrolyte refractive index was set to 1.4. The nanohole array (square lattice) has a periodicity of 300 nm. The near field is visualized at the reflection minimum (arrow), which only appears for the conventional design with WO_3 in its bright state. The left image shows the electric field and the right one shows the magnetic field. The polarization of the incident wave is along the image plane.

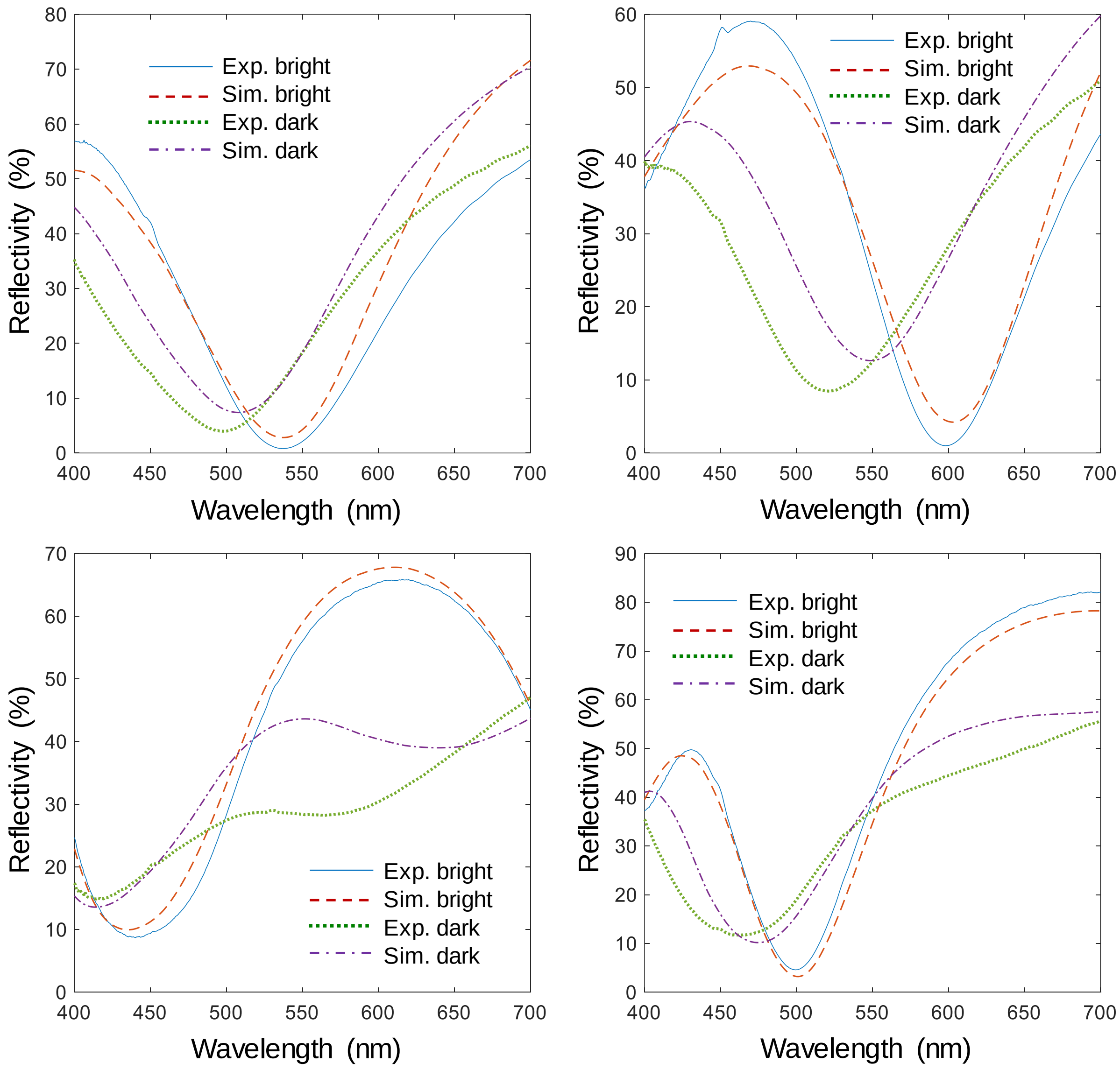

Figure S5 Comparison of Fresnel models with experimental spectra. Top left: 80 nm WO_3 (conventional). Top right: 100 nm WO_3 (conventional). Bottom left: 130 nm WO_3 (reverse). Bottom right: 140 nm WO_3 (reverse). While the permittivity was fixed to literature values, the WO_3 thickness was allowed to vary by up to 20 nm to account for uncertainty in thickness. The discrepancy between experiments and calculations is mainly attributed to small differences in the actual permittivity of our WO_3 films compared to literature values. In particular, the film morphology, stoichiometry and degree of Li^+ intercalation in the bright and dark states may vary between studies.

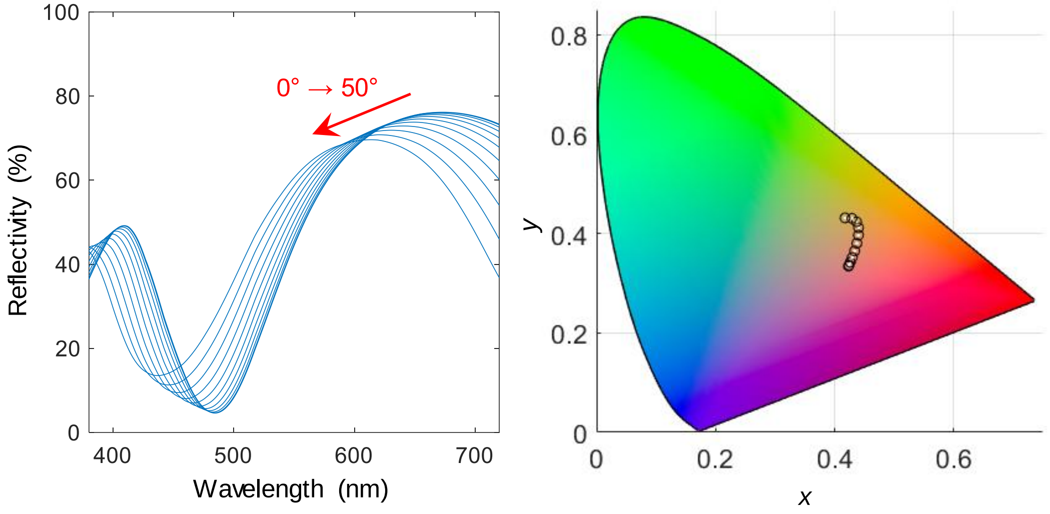

Figure S6 Simulated viewing angle dependence for a reversed sample with 150 nm WO_3, in good agreement with the supporting video. The spectra show steps of 5° and the reflectance is calculated as the average of s and p polarization. The corresponding CIE coordinates are also shown.

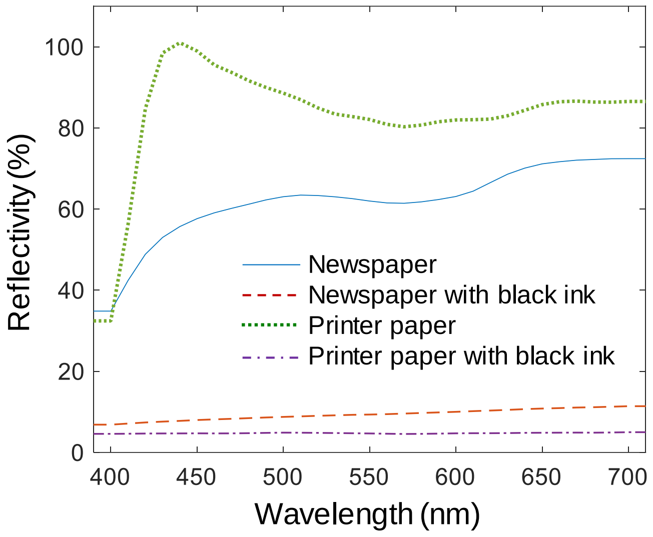

Figure S7 Reflectance spectra measured for different common papers with and without black ink. The spectra were used to calculate characteristic *Y* values mentioned in the main text. Note that the printer paper contains fluorescent compounds which makes it possible to reach values even higher than 100% since higher energy incident light is absorbed and emitted at longer wavelengths.

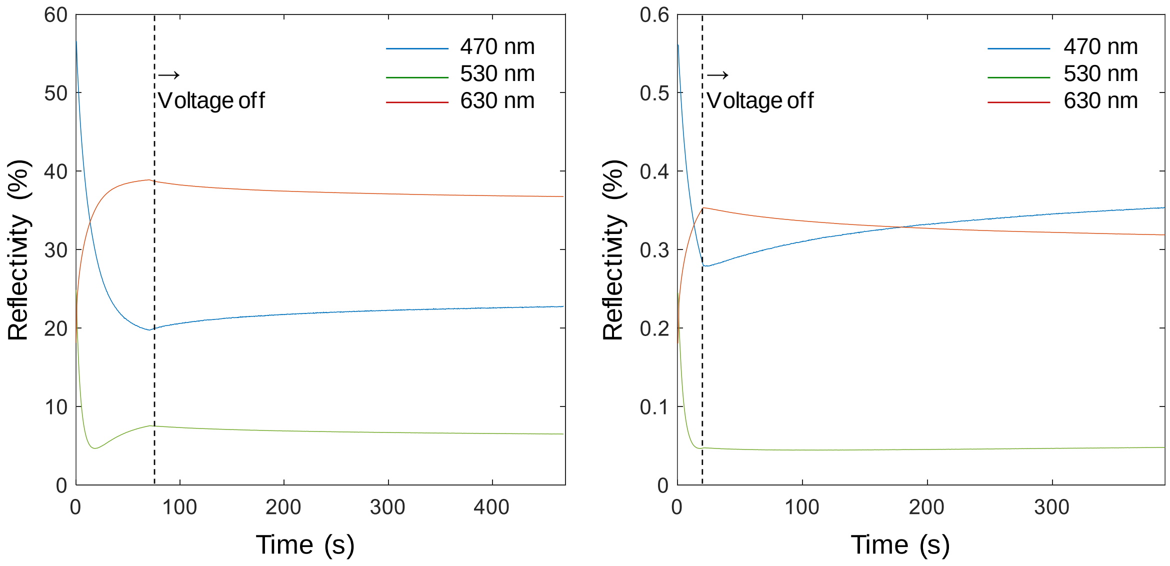

Figure S8 Example data showing switching time and bistability. (Conventional sample, 90 nm WO_3.) Left: Reflectance levels are maintained at open circuit after a complete switch (−1.5 V applied from zero until 70 s). Right: Reflectance levels change little at open circuit after a partial switch (−1.5 V applied from zero until 20 s). Note that the same voltage is used in both cases.
